# Cardiotoxicity Induced by Immune Checkpoint Inhibitors: A Pharmacovigilance Study From 2014 to 2019 Based on FAERS

**DOI:** 10.3389/fphar.2021.616505

**Published:** 2021-02-12

**Authors:** Chenxin Chen, Ting Chen, Jizhou Liang, Xiaojing Guo, Jinfang Xu, Yi Zheng, Zhijian Guo, Lijie Chi, Lianhui Wei, Xiao Chen, Xiaofei Ye, Jia He

**Affiliations:** ^1^ Department of Health Statistics, Second Military Medical University, Shanghai, China; ^2^ Department of Cardiology, Changzheng Hospital, Second Military Medical University, Shanghai, China

**Keywords:** pharmacovigilance, immune checkpoint inhibitors, cardiotoxicity, adverse drug reaction, FAERS, disproportionality analysis, reporting odds ratio, information component

## Abstract

This study was to scientifically and systematically explore the association between cardiotoxicity and immune checkpoint inhibitors (ICIs) and also to characterize the spectrum of ICI-related cardiac complications. From the first quarter of 2014 to the fourth quarter of 2019, data from the FDA Adverse Event Reporting System database were selected to conduct the disproportionality analysis. Reporting odds ratios and information components were used to evaluate the signal after statistical shrinkage transformation. In total, 7,443,137 cases and 36,326,611 drug-adverse event pairs were collected, among which 9,271 cases were identified to be related to ICI-induced cardiotoxicities. The number of male patients was much higher than that of females (5,579 vs. 3,031) and males presented a slightly higher reporting frequency than females in general, which was statistically significant (ROR = 1.04, 95%CI: 0.99–1.09, *p* < 0.001). Simultaneously, the proportion of serious or life-threatening outcomes in males was significantly higher than in females (ROR = 1.05, 95%CI: 0.96–1.15, *p* < 0.001). Importantly, ICIs were associated with over-reporting frequencies of cardiotoxicities in general (ROR025 = 1.06, IC025 = 0.08). PD-1 and PD-L1 were found to be related to cardiac adverse events, corresponding to ROR025 = 1.06, IC025 = 0.08, and ROR025 = 1.06, IC025 = 0.08, respectively, while anti-CTLA-4 (cytotoxic T-lymphocyte-associated protein 4) was significantly associated with some specific adverse events rather than common adverse events. The spectrum of cardiotoxicities induced by ICIs mostly differed among individual agents, but also demonstrated some common features. Dyspnea (*N* = 2,527, 21.25%), myocarditis (*N* = 614, 5.16%), atrial fibrillation (*N* = 576, 4.84%), cardiac failure (*N* = 476, 4.00%), and pericardial effusion (*N* = 423, 3.56%) were the top five cardiac adverse events reported in the database. Among them, myocarditis was the only one caused by all ICIs with strong signal value and high risk, warranting further attention. Overall, this investigation mainly showed the profile of cardiotoxicities caused by ICIs, which varied between different ICI therapies, but also shared some similarities in specific symptoms such as myocarditis. Therefore, it is vital and urgent to recognize and manage ICI-related cardiotoxicities, known to frequently occur in clinical practice, at the earliest point.

## Introduction

Immunotherapy was revolutionary for cancer treatment, largely improving the survival rate of patients during terminal stages of cancer. Immune checkpoint inhibitors (ICIs), including anti-CTLA-4 (cytotoxic T-lymphocyte-associated protein 4), anti-programmed cell death 1 (PD-1), and anti-programmed death ligand-1 (PD-L1), are currently used to treat various cancers, including melanoma, non-small cell lung cancer, renal cell carcinoma, and Hodgkin's lymphoma (Ribas and Wolchok, 2018; Xu et al., 2018; Khan and Gerber, 2019). However, immune-related adverse events (irAEs) are known to occur with the widespread application of ICIs, impairing several organ systems, but most commonly the gastrointestinal tract, skin, endocrine system, and liver (Costa et al., 2017; Postow et al., 2018; Wang et al., 2018). Most irAEs are manageable in the early stage, although approximately 10–17% result in fatal outcomes (Wang et al., 2018). Cardiotoxicity, especially myocarditis, has a low rate of reporting but a high rate of mortality, which may induce irreversible consequences. A large retrospective pharmacovigilance study (Wang et al., 2018) has reported that myocarditis, with only 131 cases reported, has the highest fatality rate (39.7%), whereas other common irAEs, such as endocrine events and colitis, demonstrated only 2–5% of reported fatalities. Owing to its rarity, most studies regarding cardiotoxicities are presented as case reports (Laubli et al., 2015; Johnson et al., 2016; Mahmood et al., 2018a; Hsu et al., 2018), or are only biased towards myocarditis (Heinzerling et al., 2016; Mahmood et al., 2018b; Altan et al., 2019; Chitturi et al., 2019); few studies have systematically focused on cardiotoxicity induced by ICIs. The only study (Salem et al., 2018) focusing on cardiovascular toxicities rather than cardiotoxicities detected limited potential signals. The extensive use of ICIs has increased the detection and reporting of cardiotoxicities; hence, the previous incidence might be underestimated. Given the serious outcome and increasing number of reported cases (Moslehi et al., 2018), cardiotoxicity might be a potential physiological and financial threat to patients. However, the overviewed relationship between cardiotoxicity and ICIs, as well as the spectrum of potential signals, remains unclear. In our study, we anaylzed and evaluated the association between cardiotoxicity and ICI therapies and more importantly detected and provided a comprehensive spectrum of 44 potential signals in order to provoke further attention, management, and research on this issue, as well as to serve as a clinical reference.

## Materials and Methods

### Data Sources

This real-world, retrospective study performed a disproportionality analysis based on the FDA Adverse Event Reporting System (FAERS) database, utilizing data from the first quarter of 2014 to the fourth quarter of 2019. The FAERS database is a typical spontaneous reporting system (SRS), which collects data from sources such as AE reports, medication error reports, and product quality complaints, resulting in AEs from healthcare professionals, consumers, and manufacturers.

### Procedures

Essential variables, such as PRIMARYID, CASEID, SEX, DRUGNAME, ROLE_COD, and PT (preferred terms), were extracted from different data files in the database. FAERS inevitably includes duplicate reports for receiving reports submitted by various individuals and institutions. So removal of duplicates was first conducted to reduce both false positive and false negative signals by employing a simple but widespread method called variable matching, which is used by the Medicines and Healthcare products Regulatory Agency (MHRA) and Danish Health and Medicines Authority (DHMA) (Tregunno et al., 2014). The variable matching method is the matching of key variables in two reports. If the key variables are the same, the two reports are considered duplicate reports. The key variables can be customized, but generally include report IDs, patient details (e.g., sex, birth date), suspect drug, and so on. Only the most recent report should be used, as recommended by the FDA. So, we chose PRIMARYID, CASEID, CASEVERSION, and FDA_DT as key matching variables in our study. The procedure was performed as selecting the latest FDA_DT when the PRIMARYIDs were the same, while selecting the largest CASEID and CASEVERSION when the FDA_DT and the PRIMARYID were the same. Drugs were categorized into four patterns: PS (primary suspect), SS (secondary suspect), C (concomitant), and I (interacting). Concomitant associated records were excluded to obtain better signal intensity, which is also adopted by the World Health Organization (WHO) Uppsala Monitoring Centre. As the FEARS has two variables, DRUGNAME and PROD_AI, related to medications, both generic names and brand names were used to identify ICIs in the database. The search was performed using the words ipilimumab/Yervoy, cemiplimab/ Libtayo, nivolumab/Opdivo, pembrolizumab/Keytruda, atezolizumab/Tecentriq, avelumab/Bavencio, and durvalumab/Imfinzi. All cardiac AEs in the study were coded in PTs according to MedDRA version 23.0.

### Statistical Analysis

Descriptive analysis was used to present the characteristics of all reports regarding ICI-related cardiotoxicity. Disproportionality analysis, a widely used measure in pharmacovigilance, was used to identify potential signals in our investigation. Currently, a variety of national spontaneous reporting centers are employing this method, including the WHO Monitoring Centre and the UK Yellow Card Scheme spontaneous reporting database (Montastruc et al., 2011). Reporting odds ratio (ROR) and Bayesian confidence propagation neural networks of information components (IC) are two specific indices that are calculated to detect potential associations between ICIs and cardiac AEs (Hou et al., 2014). For the sake of robustness, statistical shrinkage transformation was performed. The calculation formulas for ROR and IC after transformation are as follows (Noren et al., 2013; Zhai et al., 2019):

$$\text{ROR}=\frac{N_{\text{observed}}+0.5}{N_{\text{expected}}+0.5}$$

$$\text{IC}=\log_{2}\frac{N_{\text{observed}}+0.5}{N_{\text{expected}}+0.5}$$

$$N_{\text{expected}}=\frac{N_{\text{drug}}\times N_{\text{event}}}{N_{\text{total}}}$$

where $N_{\text{observed}}$ is the observed number of records of target drug-AEs, $N_{\text{expected}}$ is the expected number of records of target drug-AEs, $N_{\text{drug}}$ is the total number of records of the target drug, $N_{\text{event}}$ is the total number of records of target AEs, and $N_{\text{total}}$ is the total number of records in the whole database.

The calculation is based on two-by-two contingency tables. The lower limit of the 95% confidence interval for both ROR (ROR_025_) and IC (IC_025_) are criteria for a significant signal. If ROR_025_ is greater than one with at least three records or IC_025_ exceeding zero, it would be considered statistically significant and deemed a potential signal. The calculation formulas are as follows (Noren et al., 2013):

$$\text{ROR}95\%\text{CI}=e^{\ln\left(\text{ROR}\right)\pm 1.96\sqrt{\frac{1}{a}+\frac{1}{b}+\frac{1}{c}+\frac{1}{d}}}$$

$${\text{IC}}_{025}=\text{IC}-3.3\times {\left(N_{\text{observed}}+0.5\right)}^{-0.5}-2\times {\left(N_{\text{observed}}+0.5\right)}^{-1.5}$$

$${\text{IC}}_{975}=\text{IC}+2.4\times {\left(N_{\text{observed}}+0.5\right)}^{-0.5}-0.5\times {\left(N_{\text{observed}}+0.5\right)}^{-1.5}$$

ROR and IC were both calculated by comparing total or class-specific ICIs, while IC_025,_ indicating the signal intensity, was calculated in the spectrum of cardiotoxicity. All analyses were performed using SAS version 9.4 (SAS Institute Inc., Cary, NC, United States).

## Results

### Descriptive Analysis

In total, 7,443,137 records were extracted, of which 9,271 (0.125%) were reported as cardiac AEs after using ICI regimes. All demographic and clinical characteristics are presented in Table 1. Males presented a larger proportion of cardiac AEs than women. Among 9,271 cases, 5,578 were reported by male patients, accounting for 60.17%, while 3,031 (32.69%) were encountered by females. In addition, patients aged 65 years and above accounted for a greater proportion than those aged between 18 and 64 years old (46.31% vs. 33.24%), which implied that patients greater than or equal to 65 years old are more likely to be affected by ICI-induced cardiotoxicity. Most cases were reported between 2016 and 2019, with increasing tendency year by year, which is consistent with findings of Moslehi et al. (2018). It can be assumed that cardiac AEs are more likely to be observed and reported owing to the widespread use of immune therapy. Hospitalization (*N* = 3,588, 38.70%) was the most common outcome induced by ICIs, followed by death (*N* = 2,808, 30.29%). Furthermore, two high-risk outcomes, death and life-threatening event, were reported in 3,418 cases in total, accounting for 36.87%, indicating that once cardiotoxicity occurs, it is relatively easy to threaten life or directly lead to death, further confirming the high mortality rate of ICI-induced cardiotoxicity.

**TABLE 1:** Characteristics of patients with ICI-induced cardiotoxicity.

	ICIs induced cardiac AEs *n* (%)
Gender	
Male	5,578 (60.17)
Female	3,031 (32.69)
Missing or unknown	662 (7.14)
Age	
<18	17 (0.18)
18–64	3,082 (33.24)
≥65	4,293 (46.31)
Missing	1,879 (20.27)
Year	
2014	186 (2.01)
2015	469 (5.06)
2016	1,137 (12.26)
2017	1,629 (17.57)
2018	2,432 (26.23)
2019	3,418 (36.87)
Reporting countries	
America	3,520 (37.97)
Japan	1,269 (13.69)
France	899 (9.70)
Germany	667 (7.19)
Italy	366 (3.95)
Canada	338 (3.65)
Great Britain	289 (3.12)
Other countries	1,923 (20.74)
Outcome	
Death	2,808 (30.29)
Life-threatening	610 (6.58)
Disability	113 (1.22)
Hospitalization	3,588 (38.70)
Congenital anomaly	3 (0.03)
Required intervention	2 (0.02)
Other serious	1,604 (17.30)
Missing	543 (5.86)

### Signal Values Related to Different Immune Therapies

The signal values and the association between total/class-specific ICIs and cardiotoxicity are shown in Table 2. In general, ICI therapies were significantly associated with the reporting frequency of cardiac AEs (ROR_025_ = 1.06, IC_025_ = 0.08). Regarding different class-specific ICIs regimens, anti-PD-1 drugs (ROR_025_ = 1.06, IC_025_ = 0.08) and anti-PD-L1 drugs (ROR_025_ = 1.32, IC_025_ = 0.40) were also significantly associated with cardiotoxicity. Most cardiac AEs were reported in cases using anti-PD-1 drugs (*N* = 9,686, 70.91%), especially nivolumab, which presented the largest number of cardiac AE reports (*N* = 6836, 50.05%). Although anti-PD-L1 drugs are reported less frequently (*N* = 1,637, 11.98%), they demonstrated stronger signal values (ROR_025_ = 1.32, IC_025_ = 0.40); avelumab ranked second among all drugs, although it showed the strongest signal values (ROR_025_ = 1.53, IC_025_ = 0.59). In general, anti-CTLA-4 drugs did not demonstrate a significant association with cardiac AEs (ROR_025_ = 0.88, IC_025_ = −0.20); however, in our further analysis, anti-CTLA-4 drugs were significantly associated with some specific cardiac AEs, revealing markedly strong signals.

**TABLE 2:** The associations of cardiotoxicity with different ICIs regimens.

Drug	*N*	ROR	ROR_025_	ROR_975_	IC	IC_025_	IC_975_
Total ICIs	13,659	1.08	**1.06**	1.09	0.11	**0.08**	0.13
Anti-CTLA-4	2,336	0.92	0.88	0.95	−0.13	−0.20	−0.08
Ipilimumab	2,336	0.92	0.88	0.95	−0.13	−0.20	−0.08
Anti-PD-1	9,686	1.08	**1.06**	1.10	0.11	**0.08**	0.13
Nivolumab	6,836	1.13	**1.10**	1.15	0.17	**0.13**	0.20
Pembrolizumab	2,808	0.98	0.95	1.02	−0.02	−0.09	0.02
Cemiplimab	42	0.91	0.67	1.24	−0.13	−0.65	0.23
Anti-PD-L1	1,637	1.39	**1.32**	1.46	0.48	**0.40**	0.54
Atezolizumab	1,023	1.35	**1.27**	1.44	0.44	**0.33**	0.51
Avelumab	194	1.78	**1.53**	2.06	0.83	**0.59**	1.00
Durvalumab	420	1.35	**1.22**	1.49	0.43	**0.27**	0.55

The bold values mean that the values were exceeding the setting threshold, of which ROR025 was over 1 and IC025 was over 0.

### The Signal Spectrum of Cardiotoxicity Differs in Immune Therapies

**FIGURE 1:**
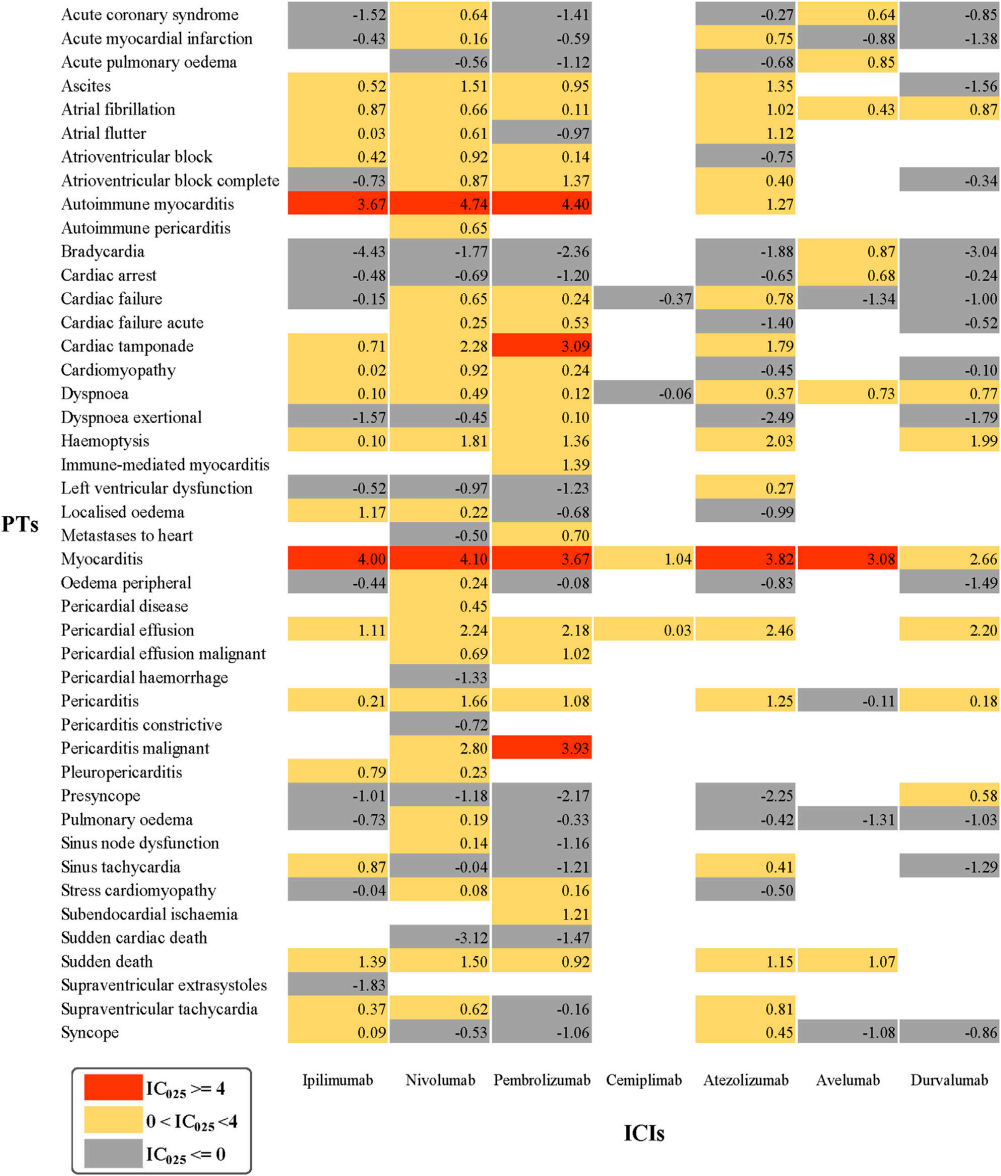
Cardiotoxicity Signal Profiles of Different ICI Strategies.

As cardiotoxicity is relatively rare and reported cases are also scarce, some important signals might be ignored if data mining is conducted only at a general level. Therefore, we performed a further exploration to determine whether there exists a connection between different ICIs and each specific cardiac AE. The cardiotoxicity signal spectrum of different ICI strategies is shown in Figure 1, where the lower limit of the 95% confidence interval of IC (IC_025_) was regarded as an indicator.

As shown in Figure 1, nivolumab presented the broadest spectrum, with a total of 29 potential ICI-induced cardiotoxicity signals detected, ranging from stress cardiomyopathy (IC_025_ = 0.08) to autoimmune myocarditis (IC_025_ = 4.74). For pembrolizumab, a total of 22 PTs as signals were observed, with signal values ranging from IC_025_ = 0.10 (Dyspnea exertional) to IC_025_ = 4.40 (autoimmune myocarditis). However, the drug with the least PTs was cemiplimab, and pericardial effusion (IC_025_ = 0.03) and myocarditis (IC_025_ = 1.04) were the only two signals detected. Interestingly, these three drugs (nivolumab, pembrolizumab, and cemiplimab) are all anti-PD-1 drugs. Cemiplimab is only used to treat patients with metastatic or locally advanced cutaneous squamous cell carcinoma (CSCC) deemed unsuitable for surgery or radiation therapy (Markham and Duggan, 2018), with the rare application resulting in a small number of reported AEs. Accordingly, cemiplimab was less analyzed and discussed in further research. For nivolumab and pembrolizumab, there were 17 PTs in common, in which autoimmune myocarditis and myocarditis were the two strongest signals, ranked first (IC_025_ = 4.74, IC_025_ = 4.40) and second (IC_025_ = 4.10, IC_025_ = 3.67), respectively. Surprisingly, pembrolizumab demonstrated the largest number of PTs, detected as signals with IC_025_ greater than or equal to 4.

Overall, 18, 8, and 7 PTs were observed to be significantly associated with anti-PD-L1 therapy involving atezolizumab, avelumab, and durvalumab, respectively. Myocarditis, dyspnea, and atrial fibrillation are three overlapping PTs. In particular, myocarditis showed the strongest signal intensity for all three anti-PD-L1 drugs (IC_025_ = 3.82, IC_025_ = 3.08, IC_025_ = 2.66). Regarding ipilimumab, the only anti-CTLA-4 drug considered in this study, although it failed to reveal a significant association with cardiac AEs initially, it produced 18 potential signals after further analysis, which simultaneously ranked the third most PTs along with atezolizumab, and overlapped in 12 PTs with nivolumab and pembrolizumab. Among the 18 signals, myocarditis was the most frequently reported; it also revealed the strongest signal strength (IC_025_ = 4.00).

Notably, myocarditis was the only AE significantly related to all seven ICIs with markedly strong intensity. Furthermore, atrial fibrillation, dyspnea, and pericardial effusion were 3 PTs detected in six ICIs. Based on the statistics performed (Table 3), dyspnea, myocarditis, atrial fibrillation, cardiac failure, and pericardial effusion were the five most common AEs significantly associated with ICI-related cardiotoxicity.

**TABLE 3:** The Five most Common ICI-related Cardiotoxicity.

Cardiac AEs	N (%)
Dyspnea	2,527 (21.25)
Myocarditis	614 (5.16)
Atrial fibrillation	576 (4.84)
Cardiac failure	476 (4.00)
Pericardial effusion	423 (3.56)

## Discussion

In patients with cancer, immunotherapy inevitably induces drug toxicity along with its actual curative effect, especially irAEs. Although the incidence is low, it can affect all tissues in the human body (Khan and Gerber, 2019). Cardiotoxicity is not a commonly observed AE; however, it can easily lead to serious outcomes, warranting further attention. Owing to the rarity of cardiotoxicity, most studies are presented as case reports (Laubli et al., 2015; Johnson et al., 2016; Mahmood et al., 2018a; Hsu et al., 2018), or only focus on a specific cardiac AE such as myocarditis (Heinzerling et al., 2016; Mahmood et al., 2018b; Altan et al., 2019; Chitturi et al., 2019), meaning cardiotoxicity lacks scientific and systematic investigations. Our research systematically conducted a data mining process employing the FAERS database and gave a overview on cardiotoxicity associated to ICIs with a wide signal spectrum, resulting in some universal conclusions. Our findings are as follows.

### The Association Between Gender and Cardiac AEs

From 2014 to 2019, the reporting rate for ICI-related cardiotoxicity was approximately 0.125%, which, to a certain extent, highlighted that cardiotoxicities induced by ICIs remain rare and the reporting rate is low. The descriptive analysis above showed that cardiac AEs were more likely to be over-reported in males than in females. After further disproportionality analysis, we observed that men had a slightly higher reporting frequency than women (ROR = 1.04, 95%CI: 0.99–1.09). Few studies have considered the gender difference in ICI-induced toxicities, and to a much lesser extent cardiotoxicity. A retrospective study based on the VigiBase has revealed that females dominate the proportion of reports in most SOCs (Watson et al., 2019), while another observational study has concluded that cardiovascular toxicities such as myocarditis, pericardial diseases, and vasculitis mainly affected male patients (Salem et al., 2018). Our results, to some extent, agree with the latter finding. This result may be attributed to the over-representation of men treated with ICIs (Conforti et al., 2018; Wu et al., 2018), but not explicitly, necessitating further evidence to verify our results.

Watson et al. have observed that although female patients reported AEs more frequently than males, male patients are more easily affected by serious or fatal AEs (Watson et al., 2019). Therefore, our study analyzed and found that 2,198 of 5,578 (39.40%) males experienced life-threatening outcomes or death; in the case of women, 1,034 of 3,031 (34.11%) demonstrated similar outcomes. This indicated that the proportion of males with serious or fatal outcomes was marginally higher than that of females (ROR = 1.05, 95%CI: 0.96–1.15).

Furthermore, some studies have concluded that the efficacy of immunotherapy varies between genders, with greater efficacy in male patients (Conforti et al., 2018; Grassadonia et al., 2018; Wu et al., 2018). These findings suggest that there might be a certain association between gender and cardiac AEs, regardless of the reporting rate, outcomes, or efficacy. Therefore, gender should be considered as a vital factor in both further studies and clinical therapy, especially in the field of cardiac irAEs.

### Different Immunotherapies Associated With Cardiotoxicity

Anti-PD-1 drugs, especially nivolumab, presented the largest number of AEs, which was consistent with the characteristics of statistical results based on VigiBase of WHO (Upadhrasta et al., 2019). In general, ICIs were significantly associated with cardiac AEs. Cardiotoxicities induced by anti-PD-1/anti-PD-L1 drugs were over-reported, but the signal intensity was weak; anti-CTLA-4 did not present a significant signal value. Previous studies have found that anti-CTLA-4 drugs demonstrate a higher number of adverse reactions than anti-PD-1/ anti-PD-L1 drugs, mostly involving the skin, gastrointestinal, and endocrine systems (Boutros et al., 2016; El Osta et al., 2017). Although it is difficult to discriminate between the AE profiles of anti-PD-1 and anti-PD-L1, anti-PD-L1 might be less toxic owing to PD-L2 signaling protecting immune homeostasis (Khoja et al., 2017; Martins et al., 2019). However, it remains to be determined whether cardiac system observations were consistent with findings in previous studies. Based on the signal spectrum in our study, we hypothesized that anti-PD-1 drugs may be more likely to cause cardiac AEs than the other two ICI regimens, with nivolumab and pembrolizumab demonstrating the broadest PTs spectrum and pembrolizumab revealing stronger signals. Furthermore, previous studies have highlighted that in terms of susceptibility to myocarditis, anti-PD-1/ anti-PD-L1 are superior to anti-CTLA-4, similar to results obtained in our study. Owing to a lack of studies on immunotherapy-induced cardiotoxicity, the rationale for weak signals and no signal for anti-CTLA-4 drugs need to be further explored and elucidated. We postulated that this may be related to the rarity of cardiotoxicity, as well as the low reporting rate.

### Myocarditis and the Signal Spectrum of Cardiotoxicity

Our research provided a comprehensive spectrum of cardiac AEs induced by different ICIs, precisely presenting indicators of irAEs in different ICI regimens. In the spectrum of cardiotoxicity, dyspnea, myocarditis, atrial fibrillation, cardiac failure, and pericardial effusion were the five most common ICI-induced cardiac AEs. Notably, myocarditis was the only strong signal significantly associated with all ICIs, including cemiplimab, revealing myocarditis as the primary focus of current immunotherapy studies.

Myocarditis is the most common and fatal cardiac AE induced by ICIs, with the infiltration of effector CD8^+^ T cells observed in myocardial biopsies (Laubli et al., 2015; Tadokoro et al., 2016; Barlesi et al., 2018; Shah et al., 2019). In total, 614 myocarditis cases were extracted in our study, and 268 (43.63%) eventually died. A previous study has reported that the fatality rate of myocarditis is approximately 27%–46% (Moslehi et al., 2018), indicating the high risk and serious consequences associated with myocarditis. Compared with anti-CTLA-4 or anti-PD-L1, anti-PD-1 showed a stronger signal value in myocarditis, especially nivolumab (IC_025_ = 4.10), which corresponded with the findings of Ji et al. (2019). Additionally, a study involving 250 reports regarding ICI-related myocarditis, also based on the FAERS database, has revealed that the number of myocarditis reports is gradually increasing, which is consistent with the overall increasing trend of ICI-related cardiac AEs observed in our study. It can be predicted that the incidence of myocarditis, as well as reporting, may increase over time. Thus, it is necessary to be vigilant for myocarditis in clinical settings with enhanced supervision, with further studies urgently needed.

### The Reasons for Higher Reporting Odds With ICIs

The cardiotoxicity of immune checkpoint inhibitors (ICIs) was first confirmed in early animal studies. The study observed that CTLA-4 and PD-1 deficient mice developed severe T cell infiltration and autoimmune dilated cardiomyopathy (Waterhouse et al., 1995). A later study (Nishimura et al., 2001) showed that ovalbumin (OVA)-specific PD-1 deficient CD8^+^ T cells were transfected into CMy-mOva mice. In these mice, OVA-specific PD-1 expressing CD8^+^ T cells can cause severe myocarditis. In addition, PD-1 deficient mice were more likely to be infected with autoimmune myocarditis than control mice by immunizing CD4^+^ T cells induced by cardiac myosin, which can cause myocarditis. Studies have shown that genetic or pharmacological depletion of PD-L1 and PD-L2 aggravates the disease severity of various autoimmune myocarditis models.

According to the analysis of drug research safety database for evaluating the frequency of cardiac irAE in patients receiving ipilimumab or ipilimumab plus nivolumab (Johnson et al., 2016), 18 of 20,594 patients (0.09%) developed myocarditis, and the incidence of myocarditis in patients receiving ipilimumab plus nivolumab was higher than that in patients receiving nivolumab alone (0.27% vs. 0.06%). In addition, patients treated with combination therapy had more serious diseases. Five of 2,974 patients treated with combination therapy died of myocarditis, while one of 17,620 patients treated with nivolumab monotherapy died. These data suggest that myocarditis is a rare disease, but dual ICI treatment increases the risk of fatal myocarditis.

In addition to myocarditis, ICIs can also induce other manifestations of cardiotoxicity (Gibson et al., 2016). One patient with metastatic lung cancer had normal ECG and cardiac catheterization results. After the second cycle of nivolumab treatment, the ECG was significantly abnormal, accompanied with right bundle branch block, and progressed to multiple ectopic beats, which eventually led to persistent ventricular tachycardia, abnormal levels of creatine kinase MB (CK-MB) and troponin I, hepatitis, and pneumonia.

The mechanism of cardiac dysfunction induced by ICIs is not completely clear, but the limited data of irAE case reports show that there are various mechanisms, including direct binding of ICI with target molecules on non-lymphocytes, inducing downstream immune activation, cross reaction between tumor antigen and target tissue, production of autoantibodies, and increase of pro-inflammatory cytokines.

### Limitations and Strengths

There are some limitations to our study. First, as a classical SRS, the FAERS database has limitations itself, with multiple data sources, a nonuniform data format, data duplication, and missing data. Second, the causal association cannot be confirmed as our study is a retrospective study. Third, the data in contingency tables were extracted as units of combination drug-AE pairs rather than reports. The two different data extraction methods may affect the results. Fourth, we only considered ICI monotherapy but failed to consider the combined use of ICIs. Nevertheless, our study quantified the potential risks scientifically and systematically with the steady support of big data, and provided a signal spectrum of cardiotoxicities induced by ICIs, which could provide valuable evidence for further studies and clinical practice in this field.

## Conclusion

With the widespread use of ICIs in the antitumor field, reports of cardiac AEs are rising, and the severity cannot be ignored. Our study, based on the FAERS database, conducted a comprehensive, retrospective analysis, exploring the relationship between ICIs and cardiotoxicity from different perspectives, as well as quantifying the potential risks, which, to some extent, can assist clinical practice, medication monitoring and management, and future investigations. Furthermore, we expect further studies focusing on ICI-induced cardiotoxicities, which could compensate for limitations and deficiencies in our study, and more comprehensively and extensively discover and confirm the risks.

## Data Availability

The raw data supporting the conclusions of this article will be made available by the authors, without undue reservation.
